# Obstetrical Memoranda

**Published:** 1857-05

**Authors:** 


*Obstetrical Memoranda. Reported by the Editor of the N. J. Medical and Surgical Reporter.*

At the January meeting of the District Medical Society for the County of Burlington, N. J., a rambling conversation occurred, in which some remarks were made by some of the members which we will endeavor to record as far as our memory will serve us.

*Ergot.*—Dr. Budd remarked that he was now attending a patient to whom he had administered ergot for the purpose of hastening the termination of a case of labor. He had never given the "confounded stuff" without regretting it afterwards. In this case the time and circumstances favored the use of the drug in an eminant degree. The os uteri was fully dilated, the presentation was natural, and nothing seemed wanting but an increase of uterine pains, which seemed to be very sluggish. Although fully borne out by the experience of others, yet, with his own former experience before his eyes, Dr. B. administered the ergot with some hesitancy, but with the desired effect of inducing uterine action This was characteristic of the action of ergot, being persistent and strong ; and there was nothing at the time that was unpleasant in the action of the drug. The labor terminated speedily and satisfactorily, but was followed by metritis, which Dr. B. feared was to be attributed to the persistent uterine action caused by the ergot. In case of the death of the patient, who was still very ill, the contemplation was to him by no means pleasant.

Dr. Gauntt understood Dr. Budd to say that some one had given the patient "No. 6" to "help the pains." He recommended Dr. B. to give the credit of the merits to the stimulating action of this medicine. Dr. G. placed great reliance on ergot, and had frequently used it with the greatest satisfaction. He often used it where others employed the forceps, and thought it preferable in most cases except primiparæ.

Dr. Coleman would be sorry to have a bad name given to ergot, for he often found it very useful, particularly, however, in cases of uterine hemorrhage. He had also used it with satisfaction, though he was understood to say that he did not use it habitually, in cases of labor.

Dr. Budd did not wish to be understood to condemn the use of the drug altogether, but it had been his experience never to use it in a case of labor without regretting it on account of its after affects. He often had recourse to warm drinks, as a cup of warm table tea, and found them to be of very great benefit in relaxing the system, inducing perspiration, and an increase flow of the vaginal secretions. Other members corroborated Dr. Budd's experience in this respect.

Dr. Stratton would not claim for rye any of the emmenagogue effects belonging to the ergot, yet he was in the habit of using rye tea, drank warm, and found it to answer a very good purpose ; and his patients often attributed great virtue to it. In many cases, it is necessary to be "doing something," be it ever so simple, in order to engage the attention of the patient, and support her flagging energies. Rye tea can do no harm, and he is not prepared to say that it does not do positive good.

Dr. Gauntt was in the habit of exhibiting ergot in combination with opium.

Dr. Stratton suggested whether the physiological action of the two drugs was not incompatible ; whether the opium would not interfere with the characteristic effect of the ergot.

Dr. Coleman thought not. He suggested that while the ergot acted on the organic nervous centres, the opium affected the nerves of sensation principally ; instancing the action of chloroform, which, while it deadens sensibility, does not interfere with the expulsive action of the uterus.

*Exhibiting Uterine Pains by Titillation.*—Dr. Butler mentioned that he had observed some time since, in a British review of an American work on Obstetrics (by Prof. Miller, of Louisville,) that the writer seemed disposed to ridicule the assertion by Prof. Miller, that uterine pains might be excited by introducing the finger within the margin of the os uteri, and passing it gently around, and by using slight traction. Dr. B. mentioned a case which recently came under his observation strongly confirmatory of Dr. Miller's suggestion. He was called to attend a woman aged about thirty-five, in her first confinement. There were two or three points of interest in the case. First, the case, although a first labor, was complicated by an extraordinary obliquity of the os uteri. It was so high on the sacrum that it was only with the greatest difficulty that it could be reached by the finger. The pains were frequent and violent, but almost entirely insufficient, from the fact that their force was expended on the anterior wall of the uterus, instead of against the os uteri. It was several hours before the os approched its normal position, in which effort he endeavored to aid nature by placing the woman on her back, by pressure on the anterior portion of the uterus, and by gentle traction on the os. In all his manipulations in this case, Dr. B. had to contend with violent, uterine pains, which were ofton excited by even the slightest contact of the finger with the os. As soon as there was sufficient dilatation of the os, attempt was made to introduce the forceps, which was only accomplished by the most patient and perserverring manipulation, on account of the extraordinary susceptibility of the os; and still more difficult was it to lock them after they were introduced, from the same cause. Pain followed the contact of the finger or blade of the forceps with the os in this case so uniformly, that the patient begged him again and again not to "make a pain" until she felt stronger.

Dr. B remarked incidentally, that after the forceps were properly locked on the head of the child, he never had a case which required the application of such powerful extractive force to effect delivery. To his mind it was a case that demonstrated the great value of the forceps.

Dr. B. had uniformly been in the habit of exciting pains by titillation, and he could not understand what spirit could have dictated the criticism referred to at the commencement of his remarks.

Dr. Stratton, and several other members, said that they were in the habit of resorting to the same means to excite uterine contraction.

Dr. Coleman said that it was well known that titillation of other sphincters and outlets of the body was followed by muscular action, and he could not see why the rule should not hold good in this instance. Titillation of the nostril excited sneezing; that of the fauces produced it of the stomach, causing vomiting; the passage of a sound or catheter through the urethra often excited the action of the bladder; and the same often held true in the case of the sphincter ani. The doctrine certainly has the support of analogy, to say nothing of experience.

*Labor retarded by an Exostosis; Laceration of Scalp.*—Dr. Cook mentioned a case of labor that came under his observation, in which he met a novel obstruction in what appeared to be an exostosis springing from one of the rami of the pubis. Delivery was accomplished with great difficulty, and not without an extensive laceration of the scalp of the child.

